# 

**Published:** 1858-10

**Authors:** 


### CONTENTS.

### ORIGINAL COMMUNICATIONS.

PAGE.

ARTICLE I.—The Annual Address delivered to the Graduates of the Atlanta Medical College, at the Commencement, Sept. 2d., 1858. By CUSTIS B. NOTTINGHAM, M. D., of Macon, Ga.......... 65

ARTICLE II.—Phenomenal History, and Post Mortem Measurements, &c., of a Hydrocephalic Subject; with some remarks upon the Etiology of Hydrocephalus. By JOHN T. BANKS, M. D., of Zebulon, Ga.......... 81

### SELECTIONS.

On the Clinical Study of the Heart-Sounds.......... 88

Dr. Lee's Reclamation for Dr. Paine.......... 97

Case of Placenta Prævia—Central Presentation—Safe Delivery.......... 109

A Point in Medical Ethics.......... 112

Biographical Sketch of the late Prof ROBERT HARE, M. D.......... 115

A Case of Puerperal Appolectic Convulsions, with Spontaneous Expulsion of the Fœtus after Death.......... 117

### EDITORIAL AND MISCELLANEOUS.

Atlanta Medical College.......... 119

Prof. Jas. B. McCaw, M. D.......... 120

Yellow Fever.......... 121

"Southern Education for Southern Youth.".......... 122

College Announcements.......... 127

Primary School of Medicine.......... 128

Payments for Journal.......... 128

